# Prevalence and Diversity of *Trypanosoma cruzi* in Triatomine Vectors and Their Blood Meal Sources from South Central Texas, USA

**DOI:** 10.3390/biology13070489

**Published:** 2024-06-30

**Authors:** Rebecca J. Kilgore, Trina Guerra, Heather Beck, Andrea Villamizar Gomez, Michael R. J. Forstner, Dittmar Hahn

**Affiliations:** 1 The Tick-Borne Disease Research Laboratory, Department of Microbiology, Immunology, and Genetics, School of Biomedical Sciences, University of North Texas Health Science Center, Fort Worth, TX 76107, USA; rebecca.kilgore@unthsc.edu; 2 Department of Biology, Texas State University, 601 University Drive, San Marcos, TX 78666, USA; tg15@txstate.edu (T.G.); heather.n.beck96@gmail.com (H.B.); avg5000@gmail.com (A.V.G.);

**Keywords:** triatomine, blood meal sources, *Trypanosoma cruzi*

## Abstract

**Simple Summary:**

Chagas disease is endemic to the state of Texas in the United States but does not have consistent surveillance or reporting. We utilized multiple sampling sites and different species of triatomine to gain data on the blood meal sources found by DNA testing for the host and vector species identities. From domestic, peridomestic, and rural sites, we found a breadth of blood meal origins including mammals, chickens, and reptiles. Unique non-native taxa utilized for blood meals enabled us to also report on extensive foraging distances for the vectors. Understanding the diversity of blood meal sources and the distances the vectors travel between meals and daytime refuges are both important aspects for understanding the spread of this disease.

**Abstract:**

The prevalence of *Trypanosoma cruzi* was assessed in 117 triatomine insects from central Texas. The *q*PCR-based results revealed *T. cruzi* in 59% of the insects (62 adults and eight nymphs), with overall prevalences of *T. cruzi* of 0% (0/9), 64% (11/17), 58% (10/17), 73% (30/41), and 57% (19/33) for the Bastrop, Caldwell, Gonzales, Guadalupe, and Hays counties, respectively. Analyses of 18S rRNA fragments confirmed *T. cuzi* in 81% of these samples. Vectors were identified as *Triatoma gerstaeckeri* (35% of which 65% were positive for *T. cruzi*), *T. sanguisuga* (21%, 43% positive), and *Paratriatoma leticularia* (0.3%, 100% positive). Food sources were recovered from 29% of the insects. Raccoons were 53% of the blood meals (83% positive for *T. cruzi*), while the remainder came from a variety of sources, including humans (33% positive), house geckos, Eastern woodrats, plain-bellied water snakes (50% positive), hispid cotton rats (0% positive), chickens (100% positive); Asian forest turtles, bison, and pigs (0% positive). The serendipitous detection of blood meal sources at known minimum distances from the collection of the vector insect enabled us to provide several instances where the insect foraging distance was greater than 400 m. These vector foraging distances are novel information that can assist in our understanding of the landscape dynamics for the spread of the pathogen.

## 1. Introduction

Chagas disease is one of many neglected tropical diseases shared across North, Central, and South America [1,2]. The causative agent of Chagas disease is *Trypanosoma cruzi,* a parasitic protozoan that has been detected in many mammalian hosts including domestic and wild animals such as rodents, opossums, raccoons, armadillos, bats, dogs, cats, goats, and pigs [2,3,4,5,6]. The parasite is naturally transmitted to mammals through infected triatomine fecal material introduced into a wound but also by oral ingestion or congenital routes [7].

In central Texas, a region with established populations of infected triatomine vectors [8,9,10,11], 70% of raccoons were found to be positive for *T. cruzi*, while other animals such as bobcats, ocelots, coyotes, and foxes revealed much a lower prevalence of 14% each [3,12]. *T. cruzi* infections were also reported from feral hogs (*Sus scrofa*), even though the prevalence was relatively low at 6% [13]. The Triatomine vector species in Texas have mainly been identified as *Triatoma gerstaeckeri* Stål 1859, though occasionally *Triatoma sanguisuga* Leconte 1855, *Paratriatoma lecticularia* Stål 1859, *Triatoma rubida* Uhler 1894, or *Triatoma protracta woodi* Uhler 1894 were observed as well [8,9,10,14]. Up to 50% and more of the insects have been reported to be infected with *T. cruzi* [8,10,14], with adult insects generally 5 to 10 times more likely to be infected than nymphs [10,15].

The diversity of potential blood meal source prey may be poorly characterized in Texas compared to the potential breadth of prey available. Similarly, while previous work has characterized the prevalence of *T. cruzi* detection in triatomines, concurrent tests for blood meal sources for the vectors are less frequently reported [8,14]. We sought to address some of these knowledge gaps for central Texas, USA. The goal of this study was to assess the prevalence of *T. cruzi* in triatomines in South Central Texas from a variety of sampling locations, characterize triatomine vectors, and subsequently determine their blood meal sources.

## 2. Materials and Methods

**Triatomine insect specimen collection and preparation.** Triatomine insects were iteratively collected opportunistically within five central Texas municipal counties (Bastrop, Caldwell, Gonzales, Guadalupe, and Hays), generally from peridomestic sites surrounding buildings and woodpiles of residential structures, except for two insects in Hays County and four insects in Caldwell County that were captured inside residences. We characterized these sites by the density of human habitation, alongside the captures (n = 6) within a home or outside it: domestic (>100 homes/km^2^), peridomestic (>10 homes/km^2^), and rural (<1/km^2^). Active search efforts were not made in non-disturbed or sylvatic environments. Additional triatomines were collected from peridomestic sites using intentionally placed wooden lumber debris piles outside a barn in Guadalupe County, Texas, or as bycatch in a sylvatic herpetofaunal study using pitfall arrays with associated bucket traps on the rural Griffith League Ranch in Bastrop County, Texas. Three adults of *Arilus cristatus* (wheel bug) and two nymphs of *Pyrrhocoris apterus* (fire bug) were used as negative controls for DNA-based analyses of *T. cruzi* and triatomine identification. Insect specimens were stored at −20 °C until extractions were performed on all available tissue recovered from the abdominal contents (about 25 mg) using the Qiagen DNeasy Blood and Tissue Kit (Qiagen, Germantown, MD, USA), following the instructions for tissue samples. DNA was resuspended in a final volume of 200 µL [8].

**Detection of *T. cruzi*.** The presence of *T. cruzi* in triatomine samples was assessed by *q*PCR targeting a 166 bp fragment of satellite DNA with primers Cruzi 1 and Cruzi 2 (Table 1) [16]. Standards used for *q*PCR reactions were PCR amplified products from *T. cruzi* ITRI/MX/99/Cari-006 originally isolated from *Triatoma picturata* and representing lineage TcI, obtained from Universidad Autonoma del Estado de Morelos, Mexico [17]. All DNA samples were diluted ten-fold for amplification and analyzed in triplicate. *q*PCRs were performed on an Eco Real-Time PCR System (Illumina, San Diego, CA, USA) in a 10 µL volume reaction with 5 µL SYBR^®^ Green (SsoAdvanced Universal SYBR^®^ Green Supermix), 0.2 µL of 10 µM Cruzi primer 1, 0.2 µL of 10 µM Cruzi 2 primer, 3.6 µL H_2_O, and 1 µL of DNA solution (Bio-Rad, Hercules, CA, USA). The *q*PCR parameters consisted of initial denaturation at 95 °C for 5 min, followed by 40 cycles at 95 °C for 15 s and 58 °C for 60 s (Table 1) [8].

**Identification of *T. cruzi*.** Samples with positive *q*PCR detection of *T. cruzi* using primers Cruzi 1 and Cruzi 2 were used to amplify a 667 bp fragment of the 18S rRNA gene of *T. cruzi* in a nested PCR, initially with primers of SSU4F and 18Sq1R (followed by using the amplicons as a template for primers SSU561F/SSU561R [19]. The initial amplicons were generated on a PTC-200 DNA Thermocycler (MJ Research, Watertown, MA, USA) of 25 µL with 1 µL of specimen sample, 12.5 µL of Green Taq polymerase, 0.8 µL of 10 µM SSU4F, 0.8 µL of 10 µM 18Sq1R, and 9.9 µL of nuclease-free H_2_O. PCR reaction parameters consisted of initial denaturation at 95 °C for 5 min, followed by 35 cycles at 95 °C for 30 s, 50 °C for 45 s, and 72 °C for 60 s (Table 1). Amplicons were cleaned using a PCR clean-up kit following standard protocols (Promega, Madison, WI, USA). The nested PCR reaction was performed with the same conditions as those listed above, with the exception of an increase in DNA template to 2 µL and a corresponding decrease in water to 8.9 µL.

Amplicons of the nested PCR were visualized on a 2% agarose gel, cleaned using Shrimp Alkaline Phosphate and Exonuclease I (Affymetrix, Santa Clara, CA, USA) following the manufacturer’s protocols, and then sequenced bidirectionally using BigDye Terminator v3.1 (Applied Biosystems, Foster City, CA, USA), with the same primers used for PCR. Sequences were analyzed on a 3500 Genetic Analyzer (Life Technologies, Carlsbad, CA, USA) and deposited at GenBank/EMBL under accession numbers MT548855-MT548904.

**Identification of Triatomines.** Triatomine insects were identified by comparative sequence analyses of partial cytochrome B or COI amplicons of mitochondrial DNA [8,27] using DNA extracts from insect intestinal samples. All COI results were obtained using identical primers and methods in our previous work [8] and utilized the same specimens. The cytochrome B amplicons reactions were performed on a PTC-200 DNA Thermocycler of 25 µL in volume with 1 µL of DNA template, 14.25 µL of nuclease-free H_2_O, 4.125 µL of 15 mg mL^−1^ BSA (Thermo Fisher Scientific, Waltham, MA, USA), 2.5 µL of 10× *Taq* Buffer, 0.5 µL of 10 µM each of 7432F forward primer and 7433R reverse primer (Table 1), 0.5 µL of 10 mM dNTP stock, 1.5 µL of 50 mM MgCl_2_, and 0.125 µL of 5 U µL^−1^ *Taq* polymerase [27]. Thermocycler parameters were 95 °C for 5 min followed by 34 cycles of 95 °C for 30 s, 45 °C for 45 s, 72 °C for 60 s, and, finally, 72 °C for 10 min [28] (Table 1). Amplicons were cleaned and sequenced bidirectionally using BigDye Terminator v3.1 and sequences were analyzed on a 3500 Genetic Analyzer as described above.

**Identification of food sources.** Blood meals of triatomines were analyzed from insect intestine samples by comparative sequence analyses of partial cytochrome B amplicons of mitochondrial DNA, with three different primer sets targeting vertebrates in general, mammals, or birds, respectively (Table 1) [22]. DNA extracted from tissue samples of bison, chicken, swallow, turkey, alligator, rat, mouse, turtle, and pig tissues were used as positive controls for these primer sets. While the primer set for mammals amplified all controls, the vertebrate primer set failed to detect turtles, and the avian primer set detected swallow, turkey, alligator, mouse, and rat but did not amplify chicken. Reactions were performed on a PTC-200 DNA Thermocycler of 25 µL with 3 µL of DNA template, 12.175 µL of nuclease-free H_2_O, 4.125 µL of 15 mg mL^−1^ BSA, 2.5 µL of 10× *Taq* Buffer, 0.5 µL of 10 µM each of forward primer and reverse primer (Table 1), 0.5 µL of 10 mM dNTP stock, 1.5 µL of 50 mM MgCl_2_, and 0.25 µL of 5 U µL^−1^ *Taq* polymerase. Amplicons were cleaned and sequenced bidirectionally using BigDye Terminator v3.1 and sequences were analyzed on a 3500 Genetic Analyzer as described above.

**Phylogenetic Analyses.** Sequences were assembled in Geneious 8.1.8 (Biomatters Ltd., Auckland, New Zealand) and checked in the GenBank/EMBL databases using the BLAST algorithm [29]. The identities and relationships among the sequences amplified were compared to available GenBank sequences using Neighbor-Joining (NJ) [30] and maximum likelihood (ML) [31] analyses, including bootstrap consensus for 1000 replicate analyses [32,33]. NJ trees used the HKY substitution method and 1000 bootstrap replicates. All trees included the percent consensus support from ML analysis where appropriate. Trees were generated to identify the strain identity of the *T. cruzi* sequences as well as phylogenetic results from the mitochondrial DNA sequences that enabled species identification of the insect vector.

## 3. Results

A total of 117 insects were classified as triatomines by sight identification, with 90 individuals characterized as adults and 27 as nymphs (Table 2). Further determination of instar stage was not performed. Morphological keys could not enable species-level determination because the frozen, individually stored specimens were in flexible plastic bags and experienced considerable damage to appendages and color changes attendant to such storage. Table 2 also includes arthropods we previously identified [8]. Those samples had been previously identified as species using the same methods as in this project but had not had blood meal assessments [8]. Those 30 insects were included in the blood meal assessments here. In 59% (70/117) of these insects (62 adults and eight nymphs), *q*PCR-based analyses indicated the presence of *T. cruzi* (Table 2). The prevalence of *T. cruzi* detected in these insects was 59% (19/33), 73% (30/41), 64% (11/17), 58% (10/17), and 0% (0/9), respectively, for the Hays, Guadalupe, Caldwell, Gonzales, and Bastrop counties (Table 2). Two of the five non-triatomines, i.e., one wheel bug and one fire bug, used as negative controls showed weak amplification, with Ct values close to the detection limit, while the remaining two wheel bugs and one fire bug did not show any amplification.

**Figure 1:**
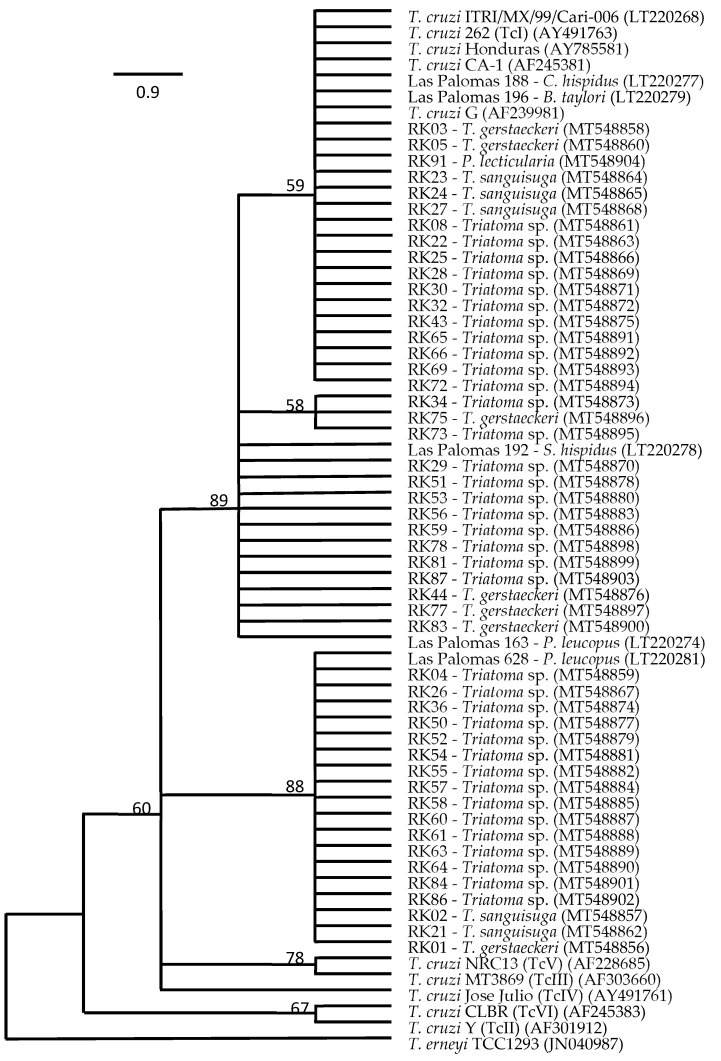
Bootstrap consensus tree of the maximum likelihood (ML) final topology (100 replicates) showing the phylogenetic relationship of *Trypanosoma cruzi* detected in intestine samples from *Triatoma gerstaeckeri*, *Triatoma sanguisuga*, *Paratriatoma lecticularia*, and unidentified *Triatoma sp*. collected in Hays, Guadalupe, and Caldwell counties, TX, USA, based on comparative sequence analysis of 18S rRNA gene fragments with those of reference isolates.

Comparative sequence analyses of a 667 bp fragment of the 18S rRNA gene targeting *T. cruzi* in a nested PCR allowed us to generate sequences from *T. cruzi* in 49 of the 70 samples, with *T. cruzi* from 31 triatomine insects from Hays, Guadalupe, and Caldwell counties clustering with representatives of Tc1 and the remaining related to Tc1, but distinct to TcII to TcVI (Figure 1). The presence of *T. cruzi* in our non-triatomine insects, i.e., the wheel bug and the fire bug, was not confirmed as *T. cruzi* by sequence data and thus was characterized as false-positive qPCR detections. Comparative sequence analyses of the 18S rRNA gene fragment targeting *T. cruzi* revealed a 98.9% pairwise identity match to *Trypanomastidae* sp. isolate PNG85 (MK929454) for the wheel bug sample and a 99.8% pairwise identity match to *Blastocrithidia papi* isolate Pa3 (KX641340) for the fire bug sample. Another sample, RK35, with a strong *q*PCR signal indicating the presence of *T. cruzi*, was also identified as a false-positive detection, with a 99.4% pairwise identity match to the same *Blastocrithidia papi* isolate Pa3 (KX641340). Table 3 provides the results for triatomine species, *T. cruzi* status, and Texas county of origin. Twenty-one of the triatomine insects positive for *T. cruzi* were identified as *Triatoma gerstaeckeri* by comparative CytB sequence analyses, with a 99.7 to 100% pairwise identity match to the sequence of specimen San Marcos I-13 (LT630441), 100% pairwise identity match to the sequence of I-JAM02 (LT630443), or a 98.2% pairwise identity match to the sequence of Parker 026 (KY305701). Twenty-five other individuals, negative for *T. cruzi*, were also identified as *T. gerstaeckeri*. Eight insects carrying *T. cruzi* and another five insects without *T. cruzi* were identified as *Triatoma sanguisuga*, with a 99.1 to 99.7% pairwise identity to the sequence of specimen PS099 (KY305708). Two adult insects, both positive for *T. cruzi*, were identified as *Paratriatoma leticularia*, with a 98.6 to 99.1% pairwise identity match to the sequence of specimen PS100 (KY305709). The remaining 39 insects positive for *T. cruzi* could not be identified at the species level and were further classified as *Triatoma* species (Figure 1).

**Figure 2:**
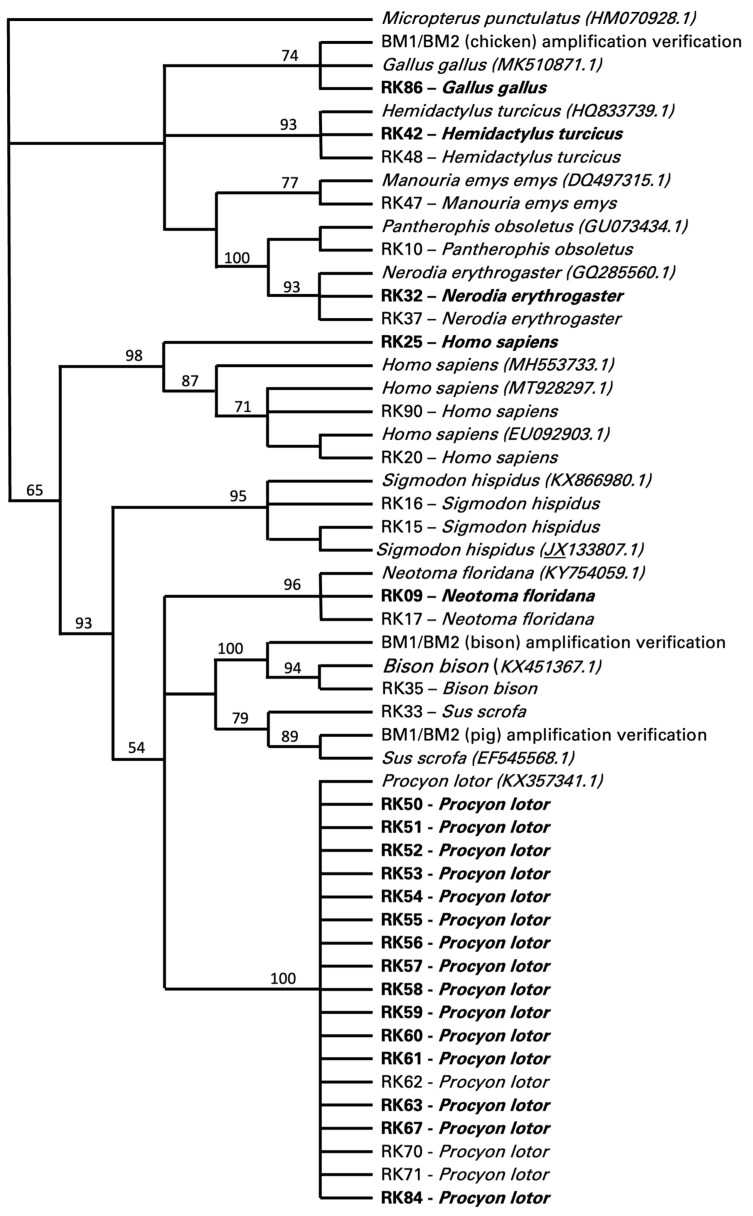
Bootstrap consensus tree of the maximum likelihood (ML) final topology identifying vertebrate blood meals of triatomines collected from Central Texas. The presence of *Trypanosoma cruzi* in triatomine samples is highlighted in bold. Sequences were obtained from PCR analysis with CytB fragments. NJ analysis with HKY substitution and 1000 bootstrap replicates was used for the final topology, with ML consensus support values included.

Food sources could only be determined in 34 of the 117 specimens, 20 of which were positive for *T. cruzi* and 14 were negative for *T. cruzi* (Figure 2). These data were all retrieved with the cytB primer set targeting vertebrates, with a few also confirmed by the cytB primer set targeting mammals. The cytB primer set designed to target only avian samples did not produce any results. The cytB primer set targeting vertebrates also retrieved non-target sequences from twenty-six insects (i.e., 23 sequences identifying *Triatoma* sp. and 3 sequences identifying *Gryllus* sp.). Of the food sources identified as vertebrates, 53% (18/34) were identified as *Procyon lotor* (raccoon), with 15 individuals positive and 3 negative for *T. cruzi* (Figure 2). Other food sources included *Homo sapiens* (human, 9%, 3/34), with one insect positive and two insects negative for *T. cruzi*; *Hemidactylus turcicus* (house gecko, 6%, 2/34), *Neotoma floridana* (Eastern woodrat, 6%, 2/34), and *Nerodia erythrogaster* (plain-bellied water snake, 6%, 2/34), all with one insect positive for *T. cruzi* and one negative; and *Sigmodon hispidus* (hispid cotton rat, 6%, 2/34), with two insects negative for *T. cruzi* (Figure 2). One insect feeding on *Gallus gallus* (chicken, 3%, 1/34) was positive for *T. cruzi*, while insects feeding on *Manouria emys emys* (Asian forest tortoise, 3%, 1/34), *Bison bison* (bison, 3%, 1/34), and *Sus scrofa* (pig, 3%, 1/34) were negative for *T. cruzi* (Figure 2).

Several of these blood meal taxa provided serendipitous information for the foraging distances of these vector insects. Specifically, the detection of these blood meals was novel among reported hosts for the vector insects, and detecting them enabled the calculation of the minimum distances between the blood meal source and the arthropod collection site using straight-line measurement. First, the Asian forest tortoise (*Manouria emys*) was captively held in an indoor/outdoor paddock. The minimum measured distance between the corner of that paddock and the insect vector collection site was 40 m. However, the tortoise could move as much as 110 m from the site of the vector. Next, a blood meal was detected from bison (*Bison bison*). These animals were held in a large, fenced facility that was a 600 m minimum distance from the insect vector location in the corner of that facility, proximal to the collection site. Importantly, this was the minimum distance, and this facility enabled the bison to travel as far as 1210 m from the site of insect collection. Finally, we also detected three instances of humans as the blood meal; one of these vector insects was also positive for *T. cruzi*. There were no human-occupied buildings, campsites, or other such occupancies near the vector collection site. The nearest potential occupied location was 430 m from the collection site, but other alternative locations were found at distances of 621–698 m (n = 5 sites). These all represent neighboring tracts of land adjacent to the insect collection location.

## 4. Discussion

*Trypanosoma cruzi* was detected in 59% of the 117 triatomine insects (76% adults and 23% nymphs), with 68% of the adults and 29% of the nymphs being positive. These results are in agreement with those of previous studies in Central Texas in which 50% or more of the triatomine insects analyzed were infected with *T. cruzi* [8,10,11,14,33]. A higher prevalence in adult insects compared to nymphs has also been reported [10,15], even though one study reported a much lower overall prevalence of 23% in adults and 4% in nymphs compared to our results [15]. High prevalence in triatomines was also found in other Texas areas [6], and the prevalence in triatomines collected in Texas was generally higher than in other states, including Oklahoma [34], Nebraska [35], or specific areas in Arizona, while other areas in Arizona were comparable to Texas values [36].

**Figure 3:**
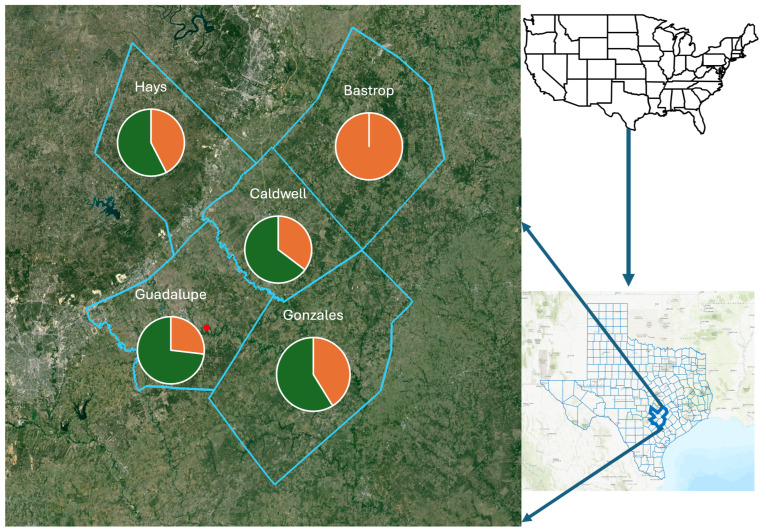
The prevalence of *Trypanosoma cruzi* detected in five counties of South Central Texas, USA. Positive detection of the pathogen (green) in vector insects varied by county, with only Bastrop County providing only negative detections (orange). The red point provides the location enabling the determination of long-distance dispersal by the vector insects.

Triatomines were predominantly collected from peridomestic habitats in five counties in South Central Texas (Figure 3), with similar prevalence values for *T. cruzi* in four counties (Hays, Guadalupe, Caldwell, Gonzales), with 57% (19/33), 73% (30/41), 64% (11/17), and 58% (10/17), respectively, while *T. cruzi* was not detected in samples from Bastrop County (0/9). While prevalence values for Hays, Guadalupe, Caldwell, and Gonzales counties represented values generally found in Texas, the lack of any detection in neighboring Bastrop County was unexpected, and most likely a consequence of the small sample size. A small sample size might also have been the cause of the low prevalence detection in triatomes from Nebraska [35], but this was not considered to explain the samples from Arizona [36]. Here, the sample size was comparable, but the sites differed with respect to the most frequently encountered triatomine species, *Triatoma rubida* and *Triatoma recurva*, respectively [36].

The presence of *T. cruzi* detected by *q*PCR-based analyses was confirmed by comparative sequence analyses of 18S rRNA gene fragments in 87% of the samples, with most of the sequences clustering with those of representatives of discrete typing unit (DTU) TcI [37] and the remaining related to TcI, but distinct from DTUs TcII to TcVI. DTU TcI is widely distributed in the Americas and found in Texas in *Triatoma* sp. from domestic and sylvatic areas [2,10,15]. Our phylogenetic analyses clearly identified members of the TcI lineage but could not explicitly characterize the remaining *T. cruzi* lineage with bootstrap support, even though underlying comparative raw sequence analyses showed a high similarity to TcIV for these isolates. Thus, while our analyses largely corroborate data on TcI lineage abundance in triatomes in Texas, the potential occurrence of TcIV in triatomines necessitates additional informative DNA sites that can enable a better-supported resolution of *T. cruzi* lineages found in our study.

Vectors mainly belonged to *Triatoma gerstaeckeri* (35% of which 65% were positive for *T. cruzi*), *Triatoma sanguisuga* (21%, 43% positive), and *Paratriatoma leticularia* (0.3%, 100% positive); however, 56% of the insects could not be identified at the species level using molecular tools. Of those identified, *T. gerstaeckeri* was confirmed as the most prominently encountered triatomine insect in Central Texas in our study, with *T. sanguisuga* and *P. lecticularia* detected much less frequently, as described in other studies [8,9,10,14,38]. Studies on triatomines in other southern states retrieved *T. rubida* and *T. recurva* (Arizona) [36], *T. sanguisuga* (Oklahoma, Nebraska) [34,35], or *T. rubida* (New Mexico, West Texas) [6] as the most commonly encountered triatomine insects, though studies often dealt with domestic rather than peridomestic or rural habitats.

**Figure 4:**
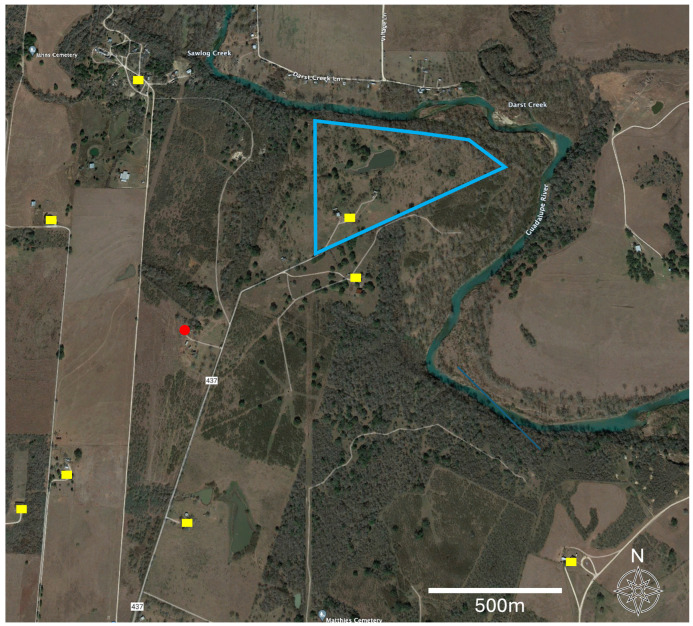
Aerial image for a portion of Guadalupe County, Texas, USA. The site context can be examined in Figure 3. The image depicts a detailed landscape context for the sampling site for Triatomine insects (red point), human-occupied domiciles (yellow), and the boundaries (blue) of an adjacent American bison paddock. The unique detection of bison among the blood meals enabled a specific minimum dispersal distance to be calculated from the collection site to the bison enclosure (600–1200 m). Similarly, the human sites also provided a set of minimum options for the distance between the arthropod detection and its nearest potential human blood meal source (>400 m).

Food sources were determined in 29% (34/117) of the insects. This was a lower blood meal recovery rate than two other studies from Texas where the recovery was 92% [9] and 63% [22]. The rate of blood meal recovery success has been shown to decline with the satiation state of the vector insect [22] and when dead arthropods as opposed to living individuals are dissected [22]. It is possible that our generally rural sites provided fewer opportunities for regular meals by the insects, decreasing their satiation and our recovery rate. All of our arthropods were frozen and not living prior to dissection. For the blood meals recovered in this study, the majority of hosts were raccoons (*P. lotor*), which were identified in 42% (15/34) of these blood meals. With 83% (15/18) of those samples being positive for *T. cruzi*, the results were higher for raccoons than those reported from other states, with 34% and 43% [39,40]. These results are closer to those obtained for Texas in other studies with prevalence values of 62% and 70% [3,41]. In Texas, DTU TcIV has been identified as the sole *T. cruzi* lineage in raccoons [3,41], with one TcI/TcIV mixed infection as an exception [3]. Comparative raw sequence analyses in our study supported these results since *T. cruzi* from raccoons showed a high similarity to sequences identifying the TcIV lineage. Blood meal sources included humans, though expectedly represented a much lower prevalence among blood meals at 9% (33% positive) in our peridomestic habitats than was detected in domestic environments, providing up to 67% [9,15,42]. We detected a blood meal from an Asian forest tortoise (*Manouria*) without *T. cruzi* infection that was held at the location but contained in an enclosure 40–110 m from the arthropod capture site. Further, we noted that for one of the peridomestic samples, while the blood meal was human, the nearest domicile occupied by people at the time of the sampling was over 400 m away (Figure 4). Finally, we also detected bison as a blood meal source though without *T. cruzi* infection. Bison were held in paddocks spanning 600–1200 m from the sampling site, indicating the high mobility of triatomine insects (Figure 4). These last two serendipitous blood meal detections uniquely enabled us to report very large distances between the source of the blood meal to the subsequent *Triatoma* sp. capture sites. These foraging distances for the insect vectors indicate that a better understanding of the insect vector movement ecology is critical to understanding the potential for exposure and the modeling of this emerging disease.

## 5. Conclusions

Our main goals for this project were to collect and identify both the triatomines potentially acting as vectors for *Trypanosoma cruzi* and further contribute to characterizing the blood sources that these insects use for meals in Texas. We were successful in triatomine sampling, with detections in all five counties in Central Texas. *Trypanosoma cruzi* was routinely detected among the potential triatomine vectors. Two species of *Triatoma* and one species of *Paratriatoma* were identified as positive for *T. cruzi*. The majority of blood meals were identified to have come from raccoons (*Procyon lotor*), but a wide array of vertebrate blood meals were confirmed including three samples of *Homo sapiens*. One of the human blood meals was also from a vector positive for *T. cruzi*. We were not able to successfully identify 44% (51/117) of the vector insects at the species level; simply storing individual specimens within freezer vials until laboratory processing would likely have prevented this difficulty.

The predominant sampling sites varied from peridomestic to rural, and the samples’ unique, non-native sources for the blood meals enabled us to infer foraging distances for the triatomine vectors. In one case this confirmed a 600 m minimum movement distance for the Triatomine from the blood meal to the daytime retreat where the insect was captured. We considered the variety of blood meal sources and the uniquely enabled forage distance measurements for the *Triatoma* sp. to be particularly relevant for modeling or assessments of the vector side of *T. cruzi* evaluations. However, these were a limited set of forage distances; moreover, they are unlikely to be the largest distances that occur. In Texas, but not only Texas, future research into these distances will be enabled because of the wide array of captive, enclosed exotic mammals and reptiles held privately across the landscape of the state. The occurrence of these non-native species could be leveraged by future research to reveal additional Triatomine dispersal/foraging data by conducting experiments collecting the vectors at known distances from unique exotic species enclosures.

## Figures and Tables

**Table 1:** Description of PCR utilized in detection of *Trypanosoma cruzi*, Triatomine species identity, and blood meal source organism.

Primers	Primer Sequence (5′–3′)	Amplicon Size	PCR Cycles	Cited
Detection of *T. cruzi* (*q*PCR)				
Cruzi 1 Cruzi 2	AST CGG CTG ATC GTT TTC GA AAT TCC TCC AAG CAG CGG ATA	166 bp	40 cycles 95 °C, 15 s 58 °C, 60 s	[16,18]
Identification of *T. cruzi* (comparative 18S rRNA gene amplicon sequence analysis)				
SSU4_F 18Sq1R	GTG CCA GCA CCC GCG GTA AT CCA CCG ACC AAA AGC GGC CA	900 bp	2 cycles each: 95 °C, 30 s 60–52 °C, 30 s 72 °C, 60 s	[19,20,21]
			followed by	
SSU561F SSU561R	TGG GAT AAC AAA GGA GCA CTG AGA CTG TAA CCT CAA AGC	650 bp	30 cycles 95 °C, 30 s 50 °C, 30 s 72 °C, 60 s	
Identification of triatomines (comparative CytB amplicon sequence analysis)				
CytB7432F CytB7433R	GGACGW GGW ATT TAT TAT GGA TC GCW CCA ATT CAR GTT AR T AA	663 bp	35 cycles 94 °C, 30 s 47 °C, 30 s 72 °C, 60 s	[22,23]
Identification of triatomine food sources (comparative CytB amplicon sequence analysis)				
BM1 BM2	CCC CTC AGA ATG ATA TTT GTC CTC A CCA TCC AAC ATC TCA GCA TGA TGA AA	358 bp	36 cycles 95 °C, 30 s 57 °C, 50 s 72 °C, 40 s	[22,24]
Mammalian a F Mammalian a R	CGA AGC TTG ATA TGA AAA ACC ATC GTT TGT AGT ART CWG GGT CHC CTA	772 bp	36 cycles 95 °C, 30 s 57 °C, 50 s 72 °C, 40 s	[22,25]
L15557 H16065	GAC TGT GAC AAA ATC CCN TTC CA GGT CTT CAT CTY HGG YTT ACA AGA C	508 bp	36 cycles 95 °C, 30 s 57 °C, 50 s 72 °C, 40 s	[22,26]

**Table 2:** Detection of *Trypanosoma cruzi* in adults and nymphs of triatomine insects collected in five counties in Central Texas, USA.

County	Total Number of Triatomines	Triatomines Negative for *T. cruzi*		Triatomines Positive for *T. cruzi*	
		Adults	Nymphs	Adults	Nymphs
Hays	33	3	11	18	1
Guadalupe	41	5	6	23	7
Caldwell	17	4	2	11	0
Gonzales	17	7	0	10	0
Bastrop	9	9	0	0	0
Total	117	28	19	62	8

**Table 3:** Triatomine species identification of *Triatoma gerstacekeri, Triatoma sanguisuga,* and *Paratriatoma lecticularia* determined by amplification and comparative analysis of mitochondrial COI or CytB gene fragments as well as *Trypanosoma cruzi* infection status and life stage of triatomines collected in Bastrop, Caldwell, Gonzales, Guadalupe, and Hays County from 2016 to 2019. Infection status was determined by the detection of pathogen DNA through *q*PCR amplification of triplicate samples. The asterisks present in the table indicate that no insect of that species and life stage was identified by molecular methods.

Species	Texas County of Capture	Negative for *T. cruzi*		Positive for *T. cruzi*	
		Adults	Nymphs	Adults	Nymphs
*Triatoma gerstaeckeri*	Bastrop	9	0	0	0
	Caldwell	3	*	3	0
	Gonzales	7	0	10	*
	Guadalupe	*	*	2	*
	Hays	*	6	5	1
*Triatoma sanguisuga*	Caldwell	1	*	1	0
	Guadalupe	1	*	4	*
	Hays	3	*	3	*
*Paratriatoma lecticularia*	Guadalupe	*	*	2	*
Triatomine	Caldwell	0	2	7	0
	Guadalupe	4	6	16	6
	Hays	*	5	9	1

## Data Availability

All sequences are available on GENBank/EMBL, as reported in the results.
